# Managing Osteoporosis and Joint Damage in Patients with Rheumatoid Arthritis: An Overview

**DOI:** 10.3390/jcm10061241

**Published:** 2021-03-17

**Authors:** Yoshiya Tanaka

**Affiliations:** The First Department of Internal Medicine, School of Medicine, University of Occupational and Environmental Health, 1-1, Iseigaoka, Kitakyushu 807-8555, Japan; tanaka@med.uoeh-u.ac.jp; Tel.: +81-93-603-1611; Fax: +81-93-691-7580

**Keywords:** rheumatoid arthritis, osteoporosis, joint destruction, DMARD, biologics

## Abstract

In rheumatoid arthritis, a representative systemic autoimmune disease, immune abnormality and accompanying persistent synovitis cause bone and cartilage destruction and systemic osteoporosis. Biologics targeting tumor necrosis factor, which plays a central role in the inflammatory process, and Janus kinase inhibitors have been introduced in the treatment of rheumatoid arthritis, making clinical remission a realistic treatment goal. These drugs can prevent structural damage to bone and cartilage. In addition, osteoporosis, caused by factors such as menopause, aging, immobility, and glucocorticoid use, can be treated with bisphosphonates and the anti-receptor activator of the nuclear factor-κB ligand antibody. An imbalance in the immune system in rheumatoid arthritis induces an imbalance in bone metabolism. However, osteoporosis and bone and cartilage destruction occur through totally different mechanisms. Understanding the mechanisms underlying osteoporosis and joint destruction in rheumatoid arthritis leads to improved care and the development of new treatments.

## 1. Introduction

Rheumatoid arthritis is a systemic autoimmune disease characterized by inflammatory polyarthritis. It occurs commonly in women in their 30s to 60s, and often causes organ disorders. In particular, persistent synovitis, caused by immune abnormality, induces abnormal bone and cartilage metabolism associated with a continuous increase in the production of inflammatory cytokines such as tumor necrosis factor (TNF) and interleukin-6 (IL-6), leading to irreversible joint destruction. The progression of joint destruction and deformity leads to irreversible physical impairment; therefore, early and appropriate treatment is necessary [1,2,3,4].

Systemic osteoporosis is caused by factors such as menopause, aging, immobility, and glucocorticoids. The incidence of osteoporosis in patients with rheumatoid arthritis is approximately two times higher than that in the general population in the same age group, and continuous glucocorticoid use is a major cause of secondary osteoporosis. However, bone and cartilage destruction and osteoporosis in rheumatoid arthritis occur through totally different mechanisms, and thus, their treatment differs.

Since the late 20th century, immunosuppressive drugs have been used to suppress undesired immune responses in the treatment of rheumatoid arthritis. The immunosuppressive drugs used in the treatment of rheumatoid arthritis are designated disease-modifying anti-rheumatic drugs (DMARDs). DMARDs are classified into two groups: synthetic DMARDs, represented by methotrexate, and biological DMARDs. The current clinical goal is to achieve remission in all patients through proper use of DMARDs. It has been reported that DMARDs are effective in preventing both structural damage to bone and cartilage and the progression of physical dysfunction [1,2,3]. Initially, glucocorticoids were used for rheumatoid arthritis; however, they are currently used just as a symptomatic therapy, only to temporarily relieve symptoms.

Osteoporosis is a highly prevalent disease characterized by a decrease in quantity and/or quality of bones, and osteoporotic fractures are the main clinical consequence, resulting in disability related to difficulty with ambulation and the performance of activities in daily life. Bisphosphonates can inhibit osteoclasts and are widely used for the treatment of primary osteoporosis and secondary osteoporosis, such as glucocorticoid-induced osteoporosis, as the most standard treatment [5,6,7]. Biologics targeting the receptor activator of the nuclear factor-κB ligand (RANKL) and sclerostin have also been introduced into the treatment of osteoporosis and have proven to be highly effective. We and others have reported that the anti-RANKL antibody was also found to be effective for the treatment of joint destruction in rheumatoid arthritis. This paper presents an overview of the management of osteoporosis and joint damage in patients with rheumatoid arthritis, based on an understanding of the underlying mechanisms.

## 2. Mechanisms of Osteoporosis and Joint Damage in Rheumatoid Arthritis

### 2.1. Osteoporosis in Rheumatoid Arthritis

**Figure 1:**
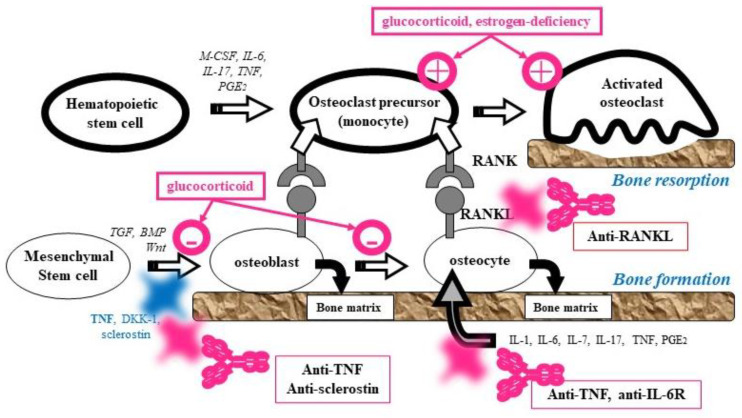
Bone formation and resorption. Osteoblasts and osteocytes differentiate from mesenchymal stem cells to produce bone matrix. Osteoclasts derived from hematopoietic stem cells mature to multinucleated osteoclasts in response to stimulation by RANKL expressed in osteoblasts and osteocytes.

Bone is an essential structure that supports the body. Bone and hard tissue protect the bodies of vertebrate animals, and bone and mineral metabolism is essential for the maintenance of life. Bone tissue is composed of bone matrix and bone cells. Osteoblasts and osteocytes differentiate from mesenchymal stem cells to produce bone matrix, which supports and maintains the bone structure. Osteoclasts are derived from hematopoietic stem cells. They mature to multinucleated osteoclasts in response to stimulation by RANKL expressed on osteoblasts and osteocytes, and become activated to induce bone resorption (Figure 1).

Bone tissue homeostasis is maintained by bone turnover (bone remodeling) via ongoing bone resorption by osteoclasts and bone formation by osteoblasts, and the regulation of the differentiation and activation of osteoblasts by osteocytes (bone modeling). Abnormalities in modeling and remodeling, caused by factors such as menopause, aging, immobility, and the use of drugs such as glucocorticoids, induce an imbalance between bone resorption and formation, and an excess of bone resorption over bone formation results in bone metabolic abnormalities—that is, osteoporosis [5,6,7]. The incidence of osteoporosis in patients with rheumatoid arthritis is approximately two times higher than that in the general population in the same age group, the risk of femoral fracture is 1.3 times higher, and that of spinal fracture is 2.4 times higher, even in patients with rheumatoid arthritis who do not use glucocorticoids [8].

Glucocorticoid-induced osteoporosis is the most common secondary cause of osteoporosis, and accounts for 25% of adverse drug reactions related to glucocorticoids. Glucocorticoids bound to receptors have anti-inflammatory and immunosuppressive effects, produced by antagonizing transcription factors such as AP-1 and NF-κB. Glucocorticoids inhibit the differentiation of mesenchymal stem cells into osteoblasts, induce apoptosis in osteoblasts and osteocytes, and decrease bone volume by suppressing the production of glycoproteins in the bone matrix. Interaction with the bone matrix is reduced by the apoptosis of osteocytes, which may lead to damage in microstructures and decreased bone quantity and quality. Glucocorticoids activate osteoclasts via secondary hyperparathyroidism and enhance the maturation and activation of osteoclasts. Thus, glucocorticoid-induced osteoporosis is caused largely by the inhibition of bone formation, damage to bone microstructures, and enhanced bone resorption, resulting in skeletal muscle degradation, leading to weakness of the limbs and hip muscles, and a decrease in the strength of bone, leading to an increased risk of falls and fractures (Figure 1) [9,10,11,12].

### 2.2. Joint Damage in Rheumatoid Arthritis

**Figure 2:**
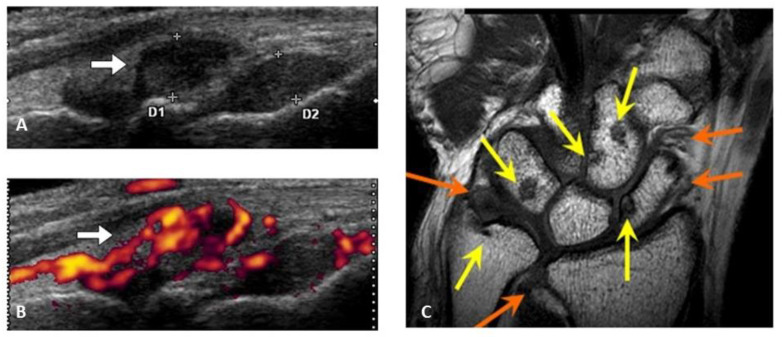
Rheumatoid synovium by ultrasonography and bone erosion by MR-imaging. Synovial hypertrophy (white bar) by systematic multiplanar grayscale ultrasonography (**A**), marked signals of synovial flow by power doppler ultrasonography (**B**), bone erosion ((**C**), yellow bar) and synovial hypertrophy ((**C**), orange bar) by MR-imaging are characteristic images in rheumatoid arthritis.

The mechanisms of bone and cartilage destruction and osteoporosis associated with rheumatoid arthritis are different, although multiple cytokine hyperactivation is involved in these two distinct pathologies. In rheumatoid arthritis, persistent stimulation by inflammatory cytokines such as TNF and IL-6 causes the formation of inflammatory synovial granulation tissue, which is characterized by the accumulation of lymphocytes, synovial proliferation, and angiogenesis (Figure 2) [13,14,15]. Matrix metalloproteinases (MMPs) are released from the granulation tissue into the joint cavity, cleave the principal components of cartilage, such as type II collagen, and diffusely degrade and resorb the cartilage from the side of the joint cavity. The serum MMP-3 level is widely used in real-world settings as an index of joint destruction [16]. As resorption progresses, cartilage gradually becomes thinner, which is shown as the joint space narrowing on radiography.

**Figure 3:**
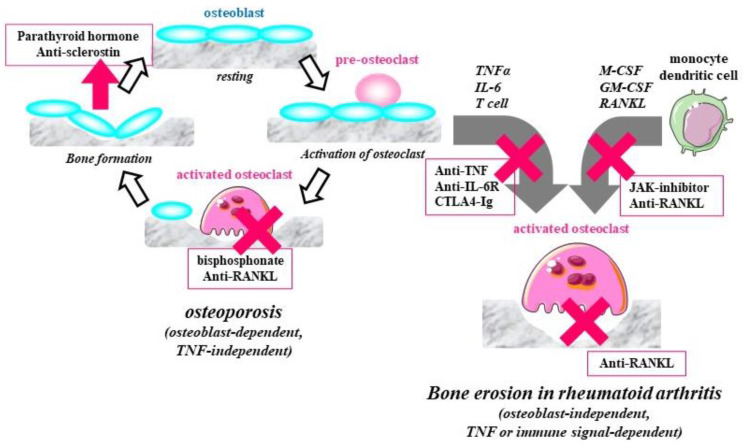
Osteoporosis and bone erosion in rheumatoid arthritis. Osteoclasts in the rheumatoid synovium deviate from normal bone metabolism by stimulation with tumor necrosis factor (TNF), IL-6, and T-cells, and cause bone destruction by the maturation and activation of osteoclasts in an osteoblast-independent manner, leading to bone erosion.

Inflammatory granulation tissue with multilayers of proliferated synovial cells grows and comes into contact with bone, where multinuclear osteoclasts are present, mainly in the contact area [12,13]. The process of the disease may extend beyond the synovium into the bone marrow, leading to osteitis. The histological findings are characterized by destruction and resorption by osteoclasts [17,18,19,20,21]. However, neither osteoblasts nor osteocytes are present in the area surrounding osteoclasts. Cytokines such as TNF and IL-6 directly stimulate the maturation of osteoclasts, and simultaneously induce the expression of RANKL in synovial cells and T cells, thus facilitating the maturation of osteoclasts, even in the absence of osteoblasts. Thus, osteoclasts in the rheumatoid synovium deviate from normal bone metabolism due to immunological stimulation with TNF, IL-6, and T-cells, and cause bone destruction by the maturation and activation of osteoclasts in an osteoblast-independent manner, leading to bone erosion, which can be identified using radiography (Figure 3) [12,22].

An imbalance in the immune system induces disorders of bone formation via the inhibition of osteoblast differentiation, and the induction of bone resorption via induction of osteoclast differentiation, leading to an imbalance in bone turnover. For example, Th1 and Th17 induce osteoclast differentiation through the production of TNF and IL-17, and Th1 cells inhibit the expression of osteoprotegerin via the production of interferon (IFN)-γ. Conversely, Treg cells inhibit the differentiation of osteoclasts via the expression of CTLA-4, and induce the migration of mesenchymal stem cells and differentiation of osteoblasts via the production of TGF-β [20,21,22]. The granulocyte macrophage colony-stimulating factor (GM-CSF) and macrophage colony-stimulating factor (M-CSF), produced by immune cells induce the differentiation of osteoclasts from not only monocytes but also dendritic cells, leading to strong bone resorption [23]. When joint destruction progresses, subluxation, dislocation, displacement, and deformation may occur, due to structural damage to the joint, the physical effects of mechanical stress, and the weakening of periarticular tissues, including tendons.

## 3. Managing Osteoporosis and Joint Damage in Rheumatoid Arthritis

### 3.1. Treatment of Joint Damage in Rheumatoid Arthritis

Before the twentieth century, controlling disease activity and preventing joint destruction were difficult tasks, and symptomatic treatment using glucocorticoids and anti-inflammatory drugs was the most commonly offered therapeutic approach. In the late 20th century, rheumatoid arthritis was recognized as an autoimmune disease, primarily characterized by arthritis, and immunosuppressive drugs were introduced to treat immune disorders and control disease activity. These drugs were called DMARDs. DMARDs are classified as follows: conventional synthetic DMARDs such as methotrexate; targeted synthetic DMARDs such as Janus kinase (JAK) inhibitors; and biological DMARDs targeting TNF, IL 6, and T cells.

In 2010, the American College of Rheumatology (ACR) and the European League Against Rheumatism (EULAR) proposed a definition of rheumatoid arthritis (chronic arthritis with joint destruction) and developed new classification criteria to differentiate it from other types of arthritis, allowing early diagnosis and treatment of rheumatoid arthritis before the development of joint destruction [24]. Methotrexate is the standard first-line therapy for rheumatoid arthritis, if there are no contraindications. Glucocorticoids are recommended just for temporary use as adjunctive therapy, only to relieve symptoms. If remission is not achieved with methotrexate for up to six months, biological DMARD or targeted synthetic DMARD should be added. Treatment is reviewed every six months until remission is achieved [25,26].

Clinical remission is defined as the lack of progression of structural damage to the joints and physical disability [27]. The Boolean remission criteria, the simplified disease activity index ≤3.3, and the clinical disease activity index ≤2.8 are used to define remission. Joint destruction in the hands, fingers, and toes is assessed using the modified total sharp score (mTSS) (joint space narrowing indicates cartilage destruction, and bone erosion indicates bone destruction) with a total score of 448 points. Structural remission was defined as a yearly mTSS of ≤0.5. With the advent of DMARDs, clinical remission has become a realistic treatment goal for all patients with rheumatoid arthritis, and now DMARDs can prevent structural damage to the joints.

### 3.2. Treatments of Osteoporosis in Rheumatoid Arthritis

Patients with rheumatic arthritis may develop osteoporosis due to menopause, aging, immobility, or glucocorticoid use. First, patients are recommended to follow the guidance to improve their lifestyles and health [28,29,30,31,32]. The main points of the guidance are as follows: reduction in risk through the improvement of daily habits such as smoking and excessive drinking; adequate understanding of their medication; vitamin D and calcium supplementation; encouragement of physical exercise such as walking; load exercise; prevention of falls; and wearing a corset while walking after a spinal fracture. In general, fractures are screened every year, and bone density is measured once every two or three years, or once every six months or one year when glucocorticoids are used.

It is recommended that patients who are diagnosed with osteoporosis based on bone density measurements receive pharmacotherapy, even if they do not have fragility fractures of the proximal femur or vertebral body [28,29,30,31,32]. Drugs for osteoporosis can be generally divided into anabolic drugs, which increase bone formation by osteoblasts, and anti-resorptive drugs, which hinder bone resorption by osteoclasts. Bisphosphonates are the standard treatment for osteoporosis. These drugs induce apoptosis of activated osteoclasts and inhibit bone resorption. In addition, denosumab, an antibody targeting RANKL, can improve the density of cancellous and cortical bone, since RANKL is essential for the maturation of osteoclasts, and is a highly effective treatment for osteoporosis. Activated vitamin D_3_ and selective estrogen receptor modulators are also used in the treatment. Parathyroid hormone (PTH) teriparatide stimulates bone formation. The PTH-related protein abaloparatide, which also has an effect on cortical bone, is now waiting for approval. As sclerostin inhibits the differentiation of osteoblasts and osteocytes, treatment with an antibody against sclerostin can inhibit bone loss (Figure 3).

Glucocorticoid-induced osteoporosis is a side effect caused by glucocorticoids, and prevention of fracture and appropriate management are needed. The ACR has developed criteria for the management of the condition according to patient characteristics, including age, sex, fracture risk, history of previous fracture, history of treatment for osteoporosis, and the dose and duration of glucocorticoids [33]. It is recommended that women aged 40 years or older, who are not expecting pregnancy, and men at moderate/high risk take oral bisphosphonate. Patients who meet at least one of the following criteria are classified as moderate/high risk:

- history of osteoporotic fracture;
- men aged 50 years or older, and women with a bone mineral density score ≤2.5;
- fracture risk assessment tool (FRAX) 10-year risk for major osteoporotic fracture ≥10% or higher;
- FRAX 10-year risk for hip fracture ≥1%;
- very high-dose glucocorticoid treatment (prednisone 30 mg/day or higher).

If oral bisphosphonates are not appropriate, injection bisphosphonates are used. If bisphosphonates are not appropriate, the PTH drug, anti-RANKL antibody, or raloxifene is used instead, in this order. Patients aged 40 years or older who discontinued glucocorticoid treatment can discontinue the drugs for osteoporosis if they are at low risk of fracture, and they are recommended to continue the drugs for osteoporosis if they are at high risk of fracture.

## 4. Treatments of Osteoporosis and Joint Damage in Rheumatoid Arthritis

### 4.1. Conventional Synthetic DMARD

Methotrexate is the most common conventional synthetic DMARD. It is the first-line standard treatment for rheumatoid arthritis if there are no contraindications [25,26]. Methotrexate exerts its anti-inflammatory effect by suppressing the proliferation of lymphocytes in the mitotic phase and synovial cells, via its antifolate activity. It indirectly reduces the destruction of bone due to cartilage resorption and osteoclast maturation by suppressing synovitis. Adverse drug reactions include liver dysfunction and gastrointestinal disorders. Bone marrow suppression, interstitial pneumonia, and infections are particularly important in the elderly. If methotrexate is not appropriate, sulfasalazine and leflunomide are recommended. Experiments in cell and animal models have shown that methotrexate induces bone turnover by decreasing the differentiation of osteoblasts, production of bone matrix, and expression of RANKL; however, the dose used in the treatment of rheumatoid arthritis has little influence on bone metabolism and the development of osteoporosis [34].

### 4.2. Biological DMARDs

Monoclonal antibodies include chimeric antibodies, humanized antibodies, human antibodies, and polyethylene glycol-modified Fab antibodies. Fusion proteins consisting of receptors and immunoglobulins are also classified as biological DMARDs. Biological DMARDs targeting TNF, IL-6 receptors, B cell antigen CD20, and T cell co-stimulation molecules are used for the treatment of rheumatoid arthritis. These drugs, in combination with methotrexate, effectively induce remission, and can prevent structural damage to the joints [1,2,3,4,25,26].

These biological DMARDs suppress synovitis, thereby producing MMP in synovial cells and releasing it into synovial fluids, and then inhibiting the degradation and resorption by MMP at the surface of the cartilage [16]. The loss of cartilage is seen as joint space narrowing on radiography. They also inhibit bone destruction by inhibiting the maturation of osteoclasts via suppression of RANKL expression in synovial fibroblasts and T cells, and by enhancement of the expression of osteoprotegerin (Figure 1 and Figure 3) [18,19,20,21,22]. They restore the capacity for differentiation into osteoblasts and the production of bone matrix by suppressing the expression of sclerostin and Dickkopf (DKK)-1. They also inhibit the production of M-CSF and GM-CSF, and inhibit differentiation from monocytes, as well as dendritic cells, into osteoclasts. Thus, it is considered that biological DMARDs inhibit bone destruction by inducing osteoblast differentiation and inhibiting osteoclast differentiation, through the correction of immunological imbalance.

Biological DMARDs can suppress the decline in bone density of the lumbar spine, femoral head, and distal radius to a certain extent, compared with monotherapy with methotrexate; however, unlike drugs for osteoporosis, biological DMARDs do not improve bone density. Unlike bisphosphonates, they do not prevent fracture [35,36].

### 4.3. Targeted Synthetic DMARDs

Biologics are administered via injection or intravenous infusion. The low-molecular weight synthetic drugs can be administered orally. They are delivered into cells through the membrane. Among the 518 kinases identified in humans, JAK is a representative tyrosine kinase. Upon binding to their receptors, cytokines activate JAK, and the activated JAK phosphorylates the receptor that binds signal transducer and activator (STAT) proteins [37,38]. Phosphorylated STAT enters the nucleus and induces transcription. Inflammatory signals are transduced through combinations of four JAK and seven STAT family members. In multiple international phase III trials, all the JAK inhibitors showed a high clinical efficacy in patients with rheumatoid arthritis who were naive to methotrexate, those who were resistant to methotrexate, and those who were resistant to biologics. The efficacy of baricitinib and upadacitinib was significantly higher than that of adalimumab, a biological DMARD targeting TNF [39,40,41,42].

**Figure 4:**
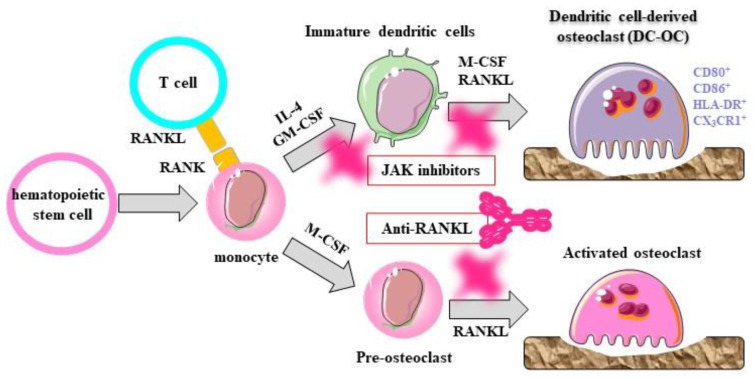
Dendritic-cell-derived osteoclasts in rheumatoid arthritis. Osteoclasts derived from dendritic cells by stimulation of GM-CSF, M-CSF, and RANKL played pathological roles in chronic inflammatory and destructive synovitis via osteoclastic bone resorption, as well as costimulatory activation of T cells.

JAK inhibitors and biological DMARDs targeting TNF show similar efficacy in inhibiting cartilage and bone destruction. In experiments using severe combined immune deficiency mice, synovium and cartilage from patients with rheumatoid arthritis were transplanted into the mice, and a JAK inhibitor was continuously infused with a pump. The treatment markedly decreased the production of human MMP-3 and human IL-6 in the transplanted synovial tissue, and completely inhibited the proliferation of the cells and their infiltration into the cartilage [43]. It has also been reported that JAK inhibitors directly or indirectly inhibit the maturation of osteoclasts by suppressing RANKL expression in synovial fibroblasts and suppressing signaling by IFNβ in dendritic cells and osteoclasts. Furthermore, JAK inhibitors are known to target signaling of multiple cytokines, including GM-CSF and M-CSF. We reported that osteoclasts derived from dendritic cells by stimulation of IL-4, GM-CSF, M-CSF, and RANKL played pathological roles in chronic inflammatory and destructive synovitis via osteoclastic bone resorption, as well as costimulatory activation of T cells (Figure 4) [23]. Such dendritic cell-derived osteoclasts could be potential targets of JAK inhibitors, which could result in the robust inhibition of bone erosion in rheumatoid arthritis. Contrarily, although few studies have reported its efficacy on osteoporosis, it can be assumed that, unlike bisphosphonates, JAK inhibitors as well as biological DMARDs do not improve bone density and do not prevent fracture [35,36].

### 4.4. Glucocorticoid

Suppression of synovitis by synthetic glucocorticoids leads to the temporal inhibition of joint destruction. However, long-term use of glucocorticoids may increase the risk of joint destruction and osteoporosis [44,45]. Glucocorticoids can induce the differentiation and activation of osteoclasts, leading to excessive bone resorption, with the bone resorption marker beginning to rise after one week [46]. DNA array analysis in rats treated with glucocorticoids showed an increase in the number of signaling molecules involved in the maturation of osteoclasts after one week. The molecules were Fcγ receptor molecules, such as M-CSF receptor c-fms, β_3_ integrin, DAP12, and c-Src [47,48]. Glucocorticoids also induce RANKL expression in osteoblasts and osteocytes and enhance the maturation of osteoclasts by suppressing the expression of osteoprotegerin as a decoy receptor of RANKL (Figure 1). Glucocorticoids promote the maturation of osteoclasts via secondary hyperparathyroidism. Furthermore, glucocorticoids induce apoptosis in osteoblasts and osteocytes, resulting in a decrease in bone volume by reducing the production of the bone matrix glycoproteins. Glucocorticoid-induced muscle weakness is a common side effect of the drug, and mainly involves weakness of the limbs and hip muscles. Glucocorticoids also decrease the strength of the bone, leading to an increased risk of falls and fractures.

### 4.5. Bisphosphonates

Bisphosphonates are the most widely used bone resorption inhibitors [28,29,30]. Bisphosphates inhibit the mevalonate pathway in osteoclasts, and inhibit bone resorption by inducing apoptosis of osteoclasts, thereby improving bone density, quality, and microstructure, and decreasing the occurrence of fracture in patients with osteoporosis. Bisphosphates are used as the first-line treatment for secondary osteoporosis, especially glucocorticoid-induced osteoporosis. Although there is a risk of jaw osteonecrosis after invasive dental treatment in patients treated with bisphosphonates and those treated with anti-RANKL antibodies, bisphosphonates are considered to be beneficial in preventing fracture [9,10,11]. Although bisphosphonates inhibit osteoclasts independent of osteoblasts, they do not have a preventive effect on bone erosion mediated by osteoclasts in altered bone metabolism in rheumatoid arthritis.

### 4.6. Anti-RANKL Antibody

RANKL, produced by osteocytes and osteoblasts, is an essential mediator of bone resorption. It activates signals mediated by RANK that are expressed on osteoclast precursor cells. An experiment with knockout mice showed that anti-RANKL antibody was associated with serious osteopetrosis and failure of tooth eruption. The anti-RANKL antibody denosumab was highly effective for the treatment of primary and secondary osteoporosis. Denosumab improved the density not only of cancellous bone, but also of all cortical bone regions, such as the distal radius, and markedly reduced the incidence of fracture of the vertebral body, non-vertebral sites, and proximal femur, compared with bisphosphonates [49]. Since the drug is distributed from the blood to the tissue without being influenced by bone microstructures, it was assumed that anti-RANKL antibody was able to gain wide access to RANKL expressed on osteocytes and osteoblasts, leading to higher efficacy in inhibiting bone resorption [50,51]. In a 10-year extension study, anti-RANKL antibody continuously improved the density of the lumbar vertebra and proximal femur, but did not increase the incidence of infections, opportunistic infections, or malignancy, confirming its long-term safety [52].

**Figure 5:**
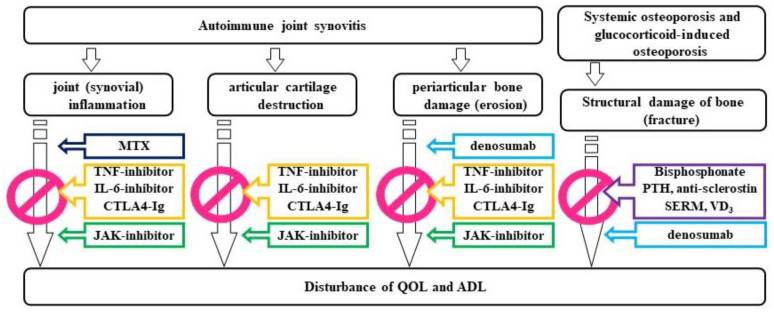
Osteoporosis, bone erosion, cartilage resorption, and synovial inflammation in rheumatoid arthritis, with their corresponding targets.

In a clinical study of denosumab for rheumatoid arthritis in Japan, treatment with denosumab did not affect the disease activity of rheumatoid arthritis, synovitis, or cartilage resorption, as reflected in the joint space narrowing score, but significantly decreased the bone erosion score compared to placebo, and also significantly decreased the levels of the bone resorption markers CTX-1 and P1NP. Anti-RANKL antibody denosumab significantly improved the bone density of the lumbar vertebra and proximal femur, and also improved glucocorticoid-induced osteoporosis [53,54,55,56]. In Japan, subcutaneous injection of denosumab 60 mg every three or six months has been approved for the prevention of progression of bone erosion associated with rheumatoid arthritis. Different from osteoporosis, bone erosion in rheumatoid arthritis is independent of osteoblasts and osteocytes (Figure 3). Biological DMARDs targeting TNF and IL-6 inhibit expression of RANKL on synovial cells and T cells, which leads to the subsequent suppression of osteoclast maturation and bone damage through RANKL, although they do not affect bone metabolism and osteoporosis. Denosumab was developed to treat systemic osteoporosis, but it can also prevent periarticular bone erosion by directly inhibiting expression of RANKL on synovial cells, which results in the inhibition of osteoclast maturation through RANKL on synovial cells [12,57]. Thus, denosumab is the only drug that has proven effective against bone destruction in rheumatoid arthritis and systemic osteoporosis, despite the different mechanisms of these conditions (Figure 5).

### 4.7. Anti-Sclerostin Antibody

Osteocytes, which account for approximately 95% of bone cells, have many dendrites, and support the skeletal framework of the body. Osteocytes play crucial roles in controlling bone modeling and remodeling. Sclerostin and DKK-1, produced primarily by osteocytes, inhibit the differentiation and activation of osteoblasts by inhibiting the Wnt signaling pathway in osteoblast precursor cells. In postmenopausal women, serum estrogen levels were negatively correlated with serum sclerostin levels, and administration of estrogen decreased serum sclerostin levels and bone resorption markers. In international clinical trials of the anti-sclerostin antibody romosozumab in postmenopausal women with osteoporosis, treatment with romosozumab significantly improved the bone density in the lumbar vertebra and the proximal femur compared to placebo, and decreased the incidence of fracture of the vertebral body and proximal femur [58,59]. In Japan, in 2019, romosozumab was approved for the treatment of patients with osteoporosis at high risk of fracture. There is no information regarding the effects of the drug on bone erosion associated with rheumatoid arthritis.

## 5. Conclusions

Osteoporosis and bone and cartilage destruction in rheumatoid arthritis occur through different mechanisms, and treatment efficacy clearly differs. Biological DMARDs targeting TNF and IL-6 inhibit synovitis and bone and cartilage destruction, but do not influence the risk of osteoporosis. However, bisphosphonates inhibit bone resorption by inducing apoptosis of osteoclasts, but are not very effective in reducing bone and cartilage destruction. Anti-RANKL antibody suppresses osteoporosis and the progression of bone erosion but is not effective for synovitis and cartilage resorption. An imbalance in the immune system may induce an imbalance in bone metabolism, which may cause structural joint damage and osteoporosis. Therefore, adequate disease control is crucial in patients with rheumatoid arthritis. Adequate and effective treatment can prevent not only destruction of bone and cartilage, but also systemic osteoporosis. There is a need to better understand the relationship between the immune system and bone metabolism. Understanding the similarities and differences between the two systems may lead to the development of new treatments.
